# Stochastic Modeling of Radiation-induced Dendritic Damage on *in silico* Mouse Hippocampal Neurons

**DOI:** 10.1038/s41598-018-23855-9

**Published:** 2018-04-03

**Authors:** Eliedonna Cacao, Vipan K. Parihar, Charles L. Limoli, Francis A. Cucinotta

**Affiliations:** 1 Department of Health Physics and Diagnostic Sciences, University of Nevada, Las Vegas, NV United States of America; 2 Department of Radiation Oncology, University of California, Irvine, CA United States of America

## Abstract

Cognitive dysfunction associated with radiotherapy for cancer treatment has been correlated to several factors, one of which is changes to the dendritic morphology of neuronal cells. Alterations in dendritic geometry and branching patterns are often accompanied by deficits that impact learning and memory. The purpose of this study is to develop a novel predictive model of neuronal dendritic damages caused by exposure to low linear energy transfer (LET) radiation, such as X-rays, γ-rays and high-energy protons. We established *in silico* representations of mouse hippocampal dentate granule cell layer (GCL) and CA1 pyramidal neurons, which are frequently examined in radiation-induced cognitive decrements. The *in silico* representations are used in a stochastic model that describes time dependent dendritic damage induced by exposure to low LET radiation. Changes in morphometric parameters, such as total dendritic length, number of branch points and branch number, including the Sholl analysis for single neurons are described by the model. Our model based predictions for different patterns of morphological changes based on energy deposition in dendritic segments (EDDS) will serve as a useful basis to compare specific patterns of morphological alterations caused by EDDS mechanisms.

## Introduction

Cranial radiotherapy is widely used to treat primary and metastatic brain tumors in children and adults, and while this can effectively extend the lifespan of cancer patients, these treatments are routinely associated with serious complications, including cognitive dysfunction^1–3^. Treatment associated neurocognitive decrements can include short- and long-term memory loss, impaired learning, attention deficits, altered spatial recognition, and deficits in multitasking and executive function^4–7^. The underlying mechanisms remain elusive; however, it is speculated to be due to the dynamic interactions between multiple cell types, including neurons, astrocytes, oligodendrocytes, microglia and endothelial cells^8^. Studies of the rodent hippocampus have correlated many of these neurocognitive sequelae to radiation-induced neuroinflammation^9^, neurogenesis impairment^9–13^ and alterations in neuronal morphology and synaptic plasticity^9,14–19^.

Radiotherapy commonly makes use of low linear energy transfer (LET) radiation, such as photons, electrons or high-energy protons with energy above about 10 MeV, with cumulative doses to the hippocampus ranging from as low as 0.1 to more than 10 Gy dependent on tumor location. Doses typically delivered to the temporal lobes occur over many fractions and vary depending on treatment plan specifics. Radiation-induced depletion of neural progenitor cells and immature neurons and changes in the neurogenic microenvironment (“niche”) define the processes that are responsible for the inhibition of neurogenesis^10,11^. Furthermore, our previous studies predicted that mouse age, type of radiation and dose-fractionation regimes are important factors in hippocampal neurogenesis impairment^20,21^. Reductions in neural stem cell proliferation and apoptosis of neural precursor cells and immature neurons in the dentate gyrus following irradiation are associated with spatial learning and memory retention deficits^12,13^. Neurogenesis generates newly born neurons that mature over the course of 4 weeks and functionally integrate into hippocampal circuitry^22,23^. However, given the relatively small percentage of functionally integrated new neurons compared to the overall hippocampal circuitry, suggests that radiation-induced changes in overt cell numbers are not likely to drive the majority of functional neurocognitive outcomes in the irradiated brain. More likely however, is that cognitive decrements are the consequence of other mechanisms, including morphological changes to mature neurons, which collectively influence the structural and synaptic plasticity of the brain. Significant dose-dependent reductions in dendritic complexity, spine density and morphology following X-rays^14^, γ-rays^15,16^ and proton irradiation^17–19^ are observed to persist for at least 30–42 days after exposure and are shown to be correlated with impairments in episodic and spatial memory retention^19^.

Dendritic arborization patterns have an impact on the function and connectivity of neurons, capable of affecting the integration of inputs and propagation of signals. Formation of the dendritic tree is driven by the dynamics of elongation, branching and retraction^24^ that include many cellular and molecular mechanisms that have been identified as regulators of dendritic growth and branching patterns^25^. Computer simulation of dendritic arborization pattern is a useful approach to discern the role of structural changes in producing functional deficits in the brain. Several mathematical and stochastic growth models have been developed to generate branching pattern variation for different types of neuron^24,26–32^. There are also existing simulation softwares^33,34^ and open-source resources^35^ that can be used to generate *in silico* neurons.

In this paper, we develop a novel predictive model that characterizes the time dependent neuronal dendritic degradation caused by exposure to low LET radiation. Computer simulated mouse hippocampal dentate granule cell layer (GCL) and CA1 pyramidal neurons, which are frequently examined in radiation-induced cognitive detriments, are first generated using simple stochastic growth models that follow the elementary rules of dendrite development^24,26,27,36,37^ and adopt specifications that manifest neuron morphometric parameters reported in rodent experimentation. We assume that energy deposition in dendritic segments (EDDS) is spatially random for low LET radiation with the number and size increasing with absorbed dose. Thus, radiation-induced changes in neuronal morphology expressed as reductions in total dendritic length, number of branch points and branch numbers can be obtained using a probabilistic model. This model is used to determine if a given branch segment would be damaged and a mathematical model of damaged segment kinetics represented by ordinary differential equations is used to determine whether the number of damaged segments would be eventually “snipped”, a term devised to distinguish this “event” from the neurobiological process of dendritic pruning. With this model, we evaluated structural changes of a single neuron. Results for a population of neurons are modeled by considering a correction for the fraction of cell loss, which increases with radiation dose.

## Results

### Computer simulated mouse hippocampal neurons

**Figure 1:**
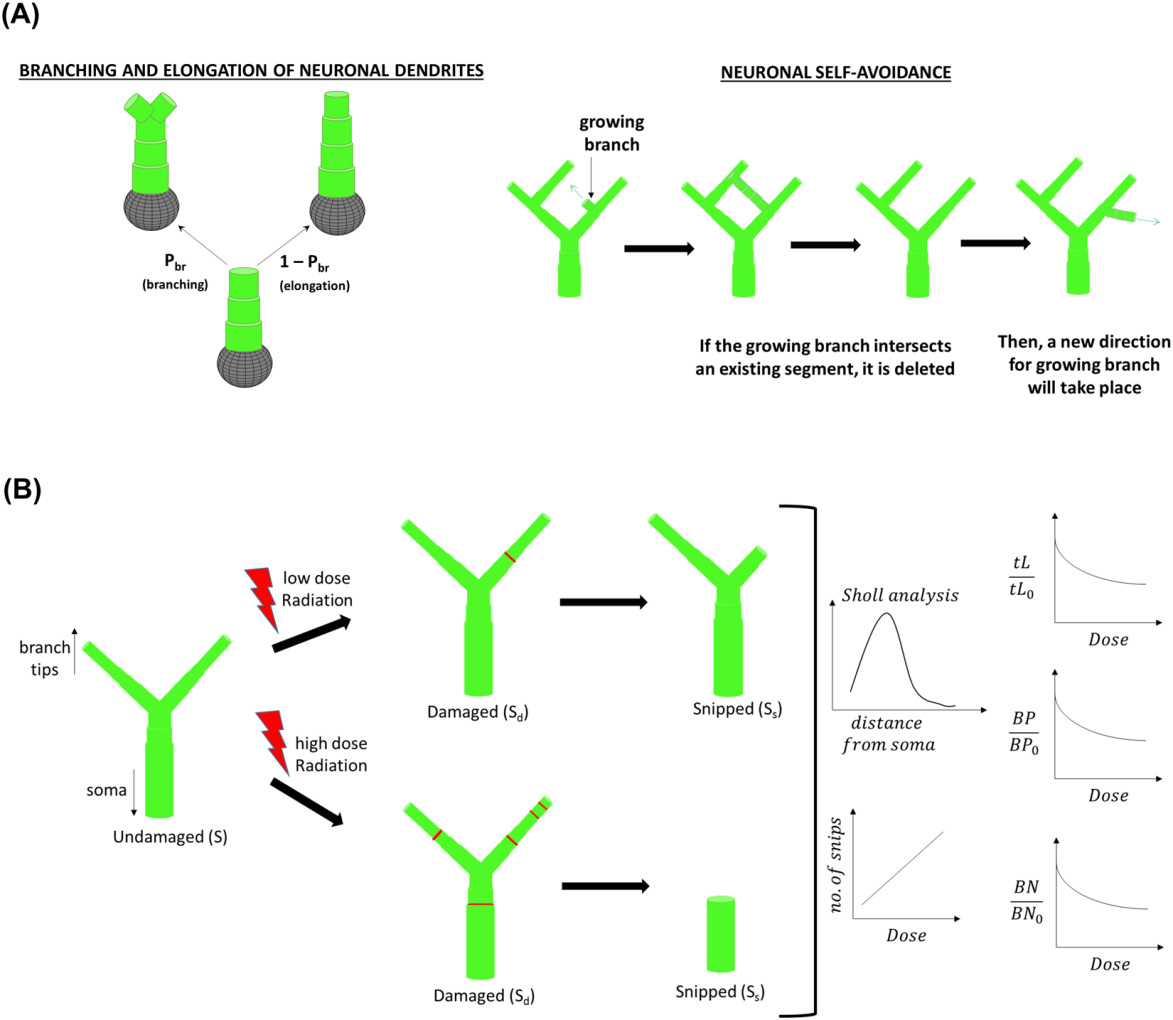
Schematic diagram of (**A**) neuronal dendritic growth model and (**B**) radiation-induced “snipping” of neuronal dendrites.

In our dendritic growth model shown in Fig. 1A, cylindrical branches are grown stochastically from the neuron cell soma. An initial segment radius of 3 μm is used and each segment step of twice the radius (cylindrical aspect ratio 1:1) can either undergo elongation or branching. Simple stochastic dendritic growth models have used different branching probabilities: a constant probability^24^, a probability as a function of branch length or the distance grown from the soma or previous branch point^26^, or a probability dependent on branch order and number of segments^28^. We adopted the branching probability as a function of branch length^26^ but used a varying parameter, α, which represents a maximum branching probability^24,28^, dependent on branch order as a means to be consistent with the reported experimental morphometric parameters in mouse hippocampal neurons. In addition, neuronal self-avoidance is considered such that when a growing segment intersects an existing branch, that growing segment is retracted back to its branch point and a new direction will be randomly selected for the growing segment.

**Figure 2:**
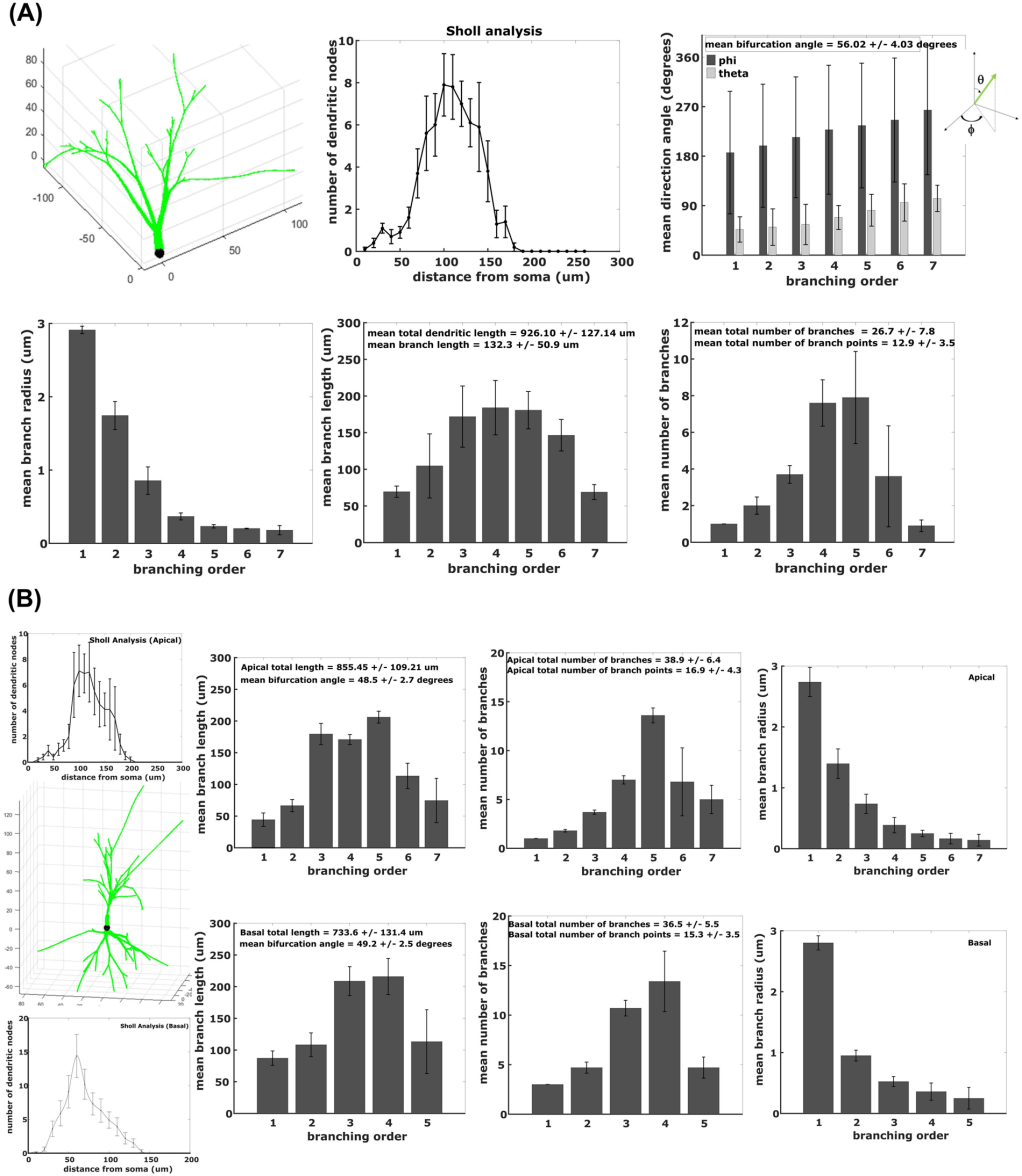
*In silico* representation of mouse hippocampal neurons: granule cell layer (GCL) neuron and CA1 pyramidal neuron. (**A**) GCL neuron parameters indicate bifurcation angle = 56.02 ± 4.03°, total dendritic length = 926.10 ± 127.14 μm, branch length = 132.3 ± 50.09 μm, branch number = 26.7 ± 7.8 and number of branch points = 12.9 ± 3.5. (**B**) CA1 pyramidal neuron parameters reveal apical total dendritic length = 855.45 ± 109.21 μm, apical bifurcation angle = 48.5 ± 2.7°, apical branch number = 38.9 ± 6.4, apical number of branch points = 16.9 ± 4.3, basal total dendritic length = 733.6 ± 131.4 μm, basal bifurcation angle = 49.2 ± 2.5°, basal branch number = 36.5 ± 5.5 and basal number of branch points = 15.3 ± 3.5. Error bars represent standard deviation from 10 neurons.

Figure 2 shows the computer simulated representations of hippocampal neurons for young adult mice (age of 1 to 4 months) along with their morphometric parameters derived from 10 generated neurons. Granule cell layer (GCL) neuron parameters in Fig. 2A indicate a mean total dendritic length of 926.10 ± 127.14 μm, mean branch length of 132.3 ± 50.9 μm, mean number of branch points of 12.9 ± 3.5, mean branch number of 26.7 ± 7.8 and mean bifurcation angle of 56.02 ± 4.03° for *in silico* neurons, which are all comparable to the reported experimental morphometric parameters in young adult mouse hippocampal granule cell neurons: total dendritic length = 1298 ± 517 μm (NeuroMorpho.org ID numbers: NMO_06175, NMO_06176)^38–40^, mean branch length = 82 ± 11 μm^38,39^, number of branch points = 7 ± 1^40^, branch number = 18 ± 5 (NeuroMorpho.org ID numbers: NMO_06175, NMO_06176)^38,39^ and mean bifurcation angle = 57.27 ± 5.70°. The graphs of branch number and mean branch length per branch order, as well as Sholl analysis, have “bell curve” shapes similar to the ones reported by Becker, *et al*.^41^.

On the other hand, Fig. 2B displays morphometric parameters for both apical and basal dendrites of CA1 pyramidal neurons. Simulated CA1 pyramidal neurons generated mean total dendritic length of 1589.1 ± 240.6 μm, mean total number of primary dendrites of 4 ± 0.2, mean branch number of 75.4 ± 11.9 and mean bifurcation angle of 48.9 ± 2.6°, which are close to parameters described in experiments: total dendritic length = 1638 ± 134 μm^42^, total number of primary dendrites = 5 ± 1^42^, mean branch number = 95.0 ± 33.9 (NeuroMorpho.org ID numbers: NMO_36622, NMO_36618) and mean bifurcation angle = 51.9 ± 0.6° (NeuroMorpho.org ID numbers: NMO_36622, NMO_36618)^42^. Moreover, computer simulated neurons have mean apical total dendritic length of 855.45 ± 109.21 μm, mean apical primary dendrite of 1 ± 0.1, mean basal total dendritic length of 733.6 ± 131.4 μm and mean basal primary dendrite of 3 ± 0.1, which are similar to experimental values of 896 ± 66 μm, 1.1 ± 0.09, 742 ± 68 μm and 3.6 ± 0.2, respectively^42^. Sholl analysis of apical and basal dendrites of *in silico* CA1 pyramidal neurons are also analogous to experimentally derived Sholl analysis^19^ where maximum dendritic intersections are found at approximately 100 μm from the soma for apical dendrites and at approximately 50 μm from the soma for basal dendrites.

### Radiation-induced alterations in neuronal dendritic structure

Dendritic damages caused by exposure to low LET radiation are conveyed by Sholl analysis. Our model considers the spatial dependence of the snips for a given radiation dose and Monte-Carlo trial, which lead to predictions of the reductions in total dendritic length, number of branch points and branch numbers. For a dendritic branch with more than one snip site, surviving segments and end-point branches are determined by the snipped-segments closest to the soma on the tip-to-soma direction pathway, as illustrated in Fig. 1B.

Our model of radiation-induced dendritic damage has two components: (1) a probabilistic model that evaluates if a given branch segment would be damaged by radiation exposure based on the EDDS, and (2) a mathematical model of damaged segment kinetics. For the first component, every segment in each dendritic branch of the computer simulated neuron is assessed if it is damaged using a probability function that is dependent on the EDDS and neuronal segment volume, such that high radiation dose and small segment volume would result in a high damage probability. We define a parameter D_d_ that represents a characteristic dose where 37% of the segments are undamaged and is a function of segment volume defined by the Hill-type equation. Supplementary Figure S1 shows the effects of varying different parameters on D_d_ and damage probability (P_d_). We decided to utilize a Hill function apparent constant of K = 0.01 because this value gives a varying radiosensitivity for a 0.2 μm to 0.5 μm segment radius that corresponds to the 4^th^–7^th^ branch order and a constant radiosensitivity for segments found in the 1^st^–3^rd^ branch order. Other Hill function apparent constants, D_m_ and Hill coefficient (η), are selected based on the value that provides the best fit with the experimental data (as illustrated in Supplementary Figure S2). Table 1 shows the summary of parameters used for both GCL and CA1 pyramidal neurons (apical and basal).

**Table 1:** Parameters for mouse hippocampal neurons after exposure to low-LET radiation.

Parameters	Granule cell layer neurons		CA1 pyramidal neurons	
	Gamma rays	Protons	Protons	
			Apical	Basal
K	0.01	0.01	0.01	0.01
D_m_ (Gy)	3000	2000	2000	2000
Hill coeff, η	3.5	3	2	3.5
D_0_ (Gy)	25	18	—	—

For the second component of the model, the kinetics of radiation-induced damaged segments is described using a stochastic solution to ordinary differential equations which describe that each damaged segment can either be repaired or snipped. Supplementary Figure S3(A) displays a sample graph showing the number of undamaged, damaged and snipped segments as a function of post irradiation time. We first defined the snip reaction rate constant (α_S_) as a function of radiation dose (refer to Supplementary Figure S3(B)). The linear quadratic dose function was then selected because we assumed that all damaged segments would be repaired or snipped at about 30 days after radiation exposure and that there should be a significant difference between 10 days and 30 days post exposure time for a 10 Gy radiation dose based on experimental obsevations^15,17^. In Supplementary Figure S3(C), we assumed that the repair reaction rate constant (α_R_) is a fraction of α_S_. We decided to use α_R_ = 0.5*α_S_ since it leads to a plausible number of repaired (included in undamaged) and snipped number of segments.

**Figure 3:**
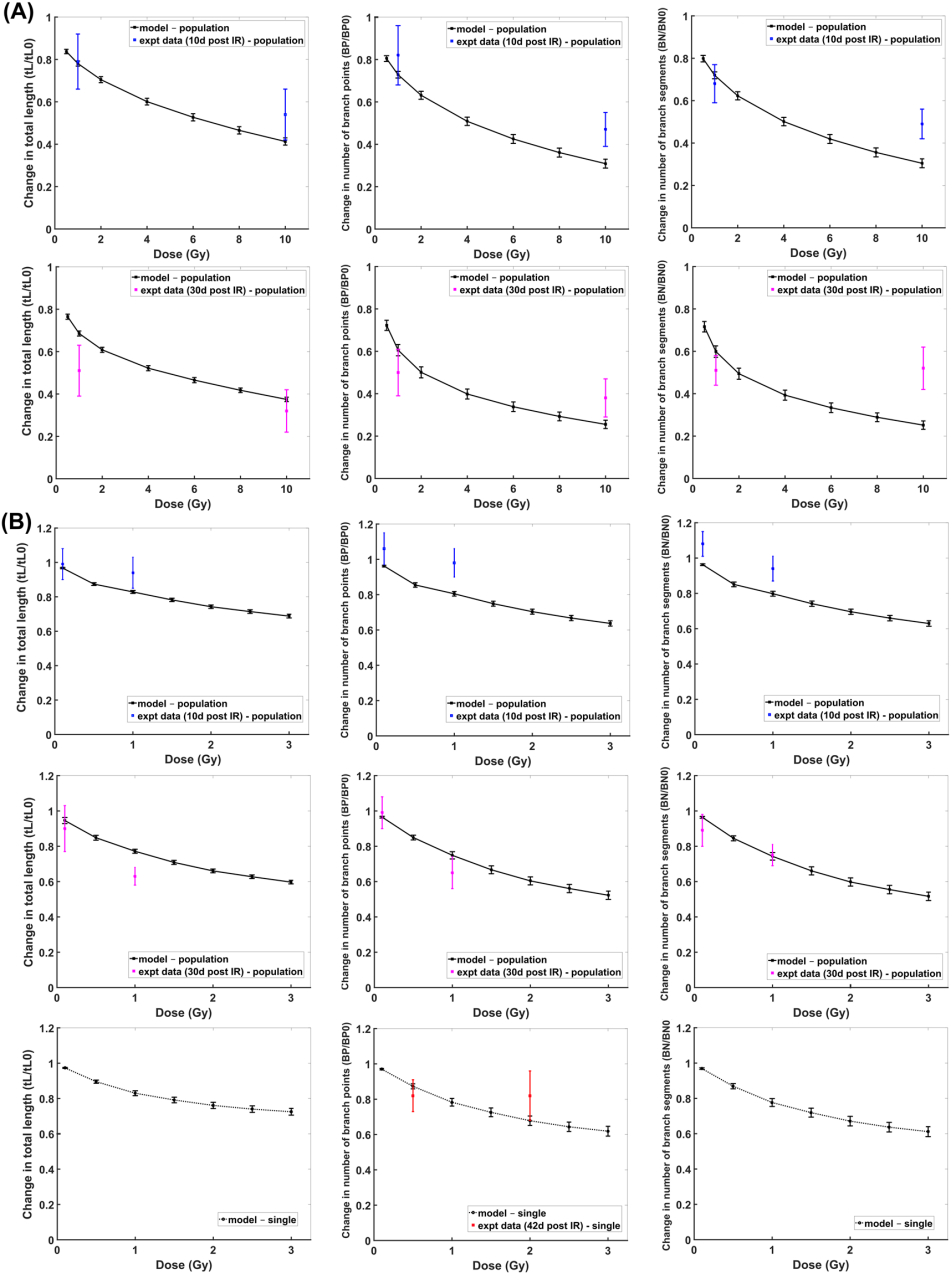
Comparison of modeling results with experimental data: Dose-dependent GCL neuron dendritic damage induced by gamma rays (**A**) and proton radiation (**B**) at 10 days (blue), 30 days (magenta) and 42 days (red) post exposure (solid line represent damage on population of neurons while dashed line represent damage on single neuron; error bar represents standard error of the mean for both modeling and experimental results).

**Figure 4:**
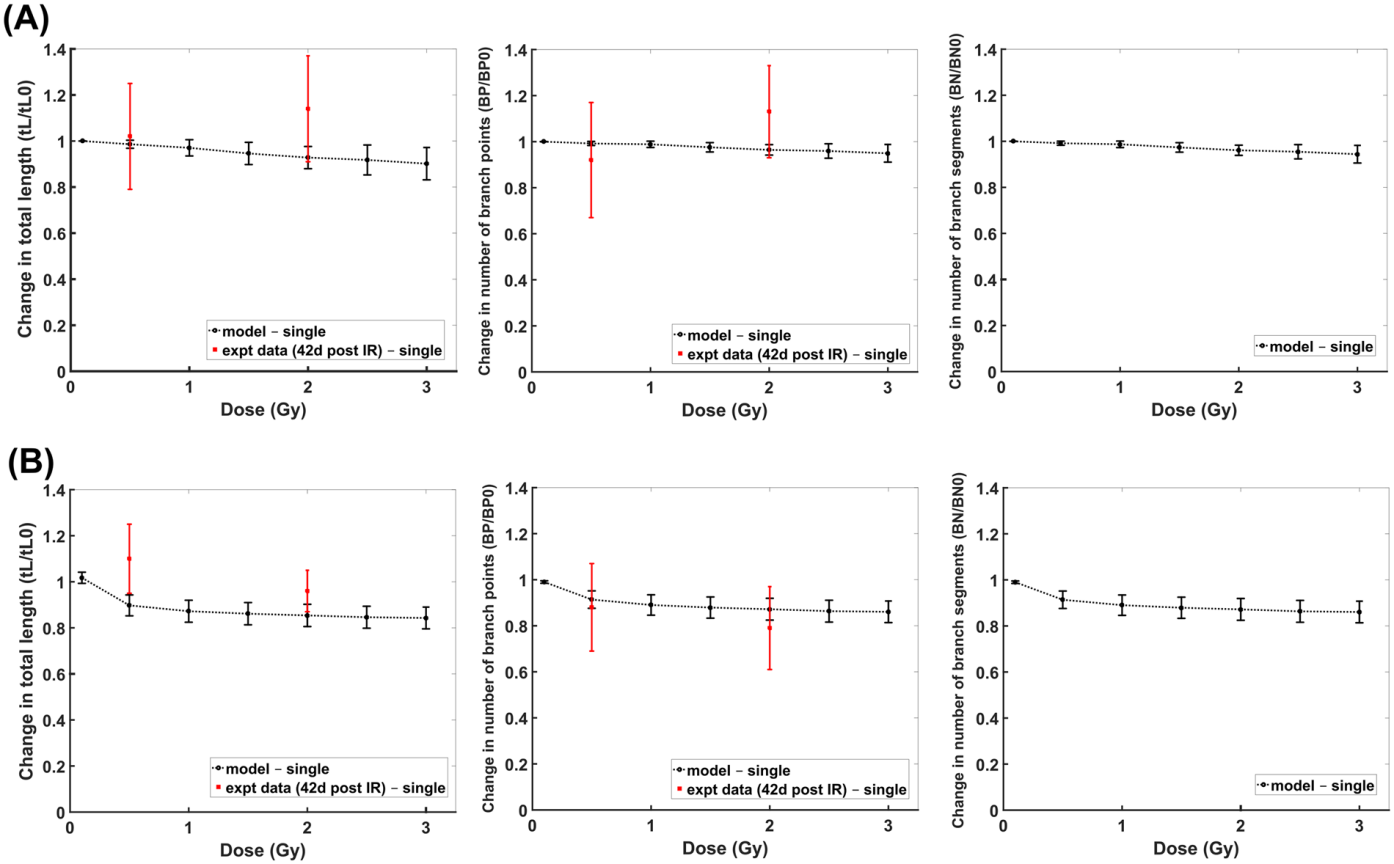
Comparison of modeling results with experimental data: Dose-dependent dendritic damage induced by proton radiation on apical (**A**) and basal (**B**) dendritic branches of CA1 pyramidal neuron at 42 days after irradiation (error bar represents standard error of the mean for both modeling and experimental results).

Comparison of our modeling results with the experimental data for granule cell layer neurons are shown in Fig. 3. Modeling results of dendritic damages induced by γ-rays (Fig. 3(A)) and by proton irradiation (Fig. 3(B)) at 10 days and 30 days post exposure times are comparable to the reported experimental data^15,17^, measured from thin slices of brain tissue that contain populations of neurons. Experimental observations from slices of brain tissue should consider differences in the number of cells observed between controls and irradiated tissues due to irradiation induced apoptosis. To translate our modeling results of dendritic structural changes from a single neuron to populations of neurons, we estimated characteristic doses for cell losses of D_0_ = 25 Gy for γ-rays and D_0_ = 18 Gy for proton radiation. These values are evaluated from reported experimental data^43–45^ but we also consider that neuron death could be via soma death, excessive dendritic branch snipping (maybe parallel to growth cone collapse) or other forms of apoptosis and/or autophagy. Reported experimental data for neuron death is either expressed by soma death evaluated by DAPI staining and TUNEL assay^43^ or by excessive dendritic branch snipping which is maybe parallel to growth cone collapse that leads to apoptosis^44,45^. Our estimated values of D_0_ for γ-rays and proton ion beams are interpolated based on these reported experimental data for X-rays and carbon ion beams^43–45^ and relative biological effectiveness of different radiation quality. On the contrary, experimental data of proton radiation-induced damages at 42 days post exposure time are measured from a single neuron using Golgi staining^19^ and are simulated by our model. In Fig. 4, our modeling results for CA1 pyramidal neurons are compared with the reported experimental data obtained by imaging single neurons^19^. Both apical and basal dendritic damages acquired by our model are similar to the experimental results.

**Figure 5:**
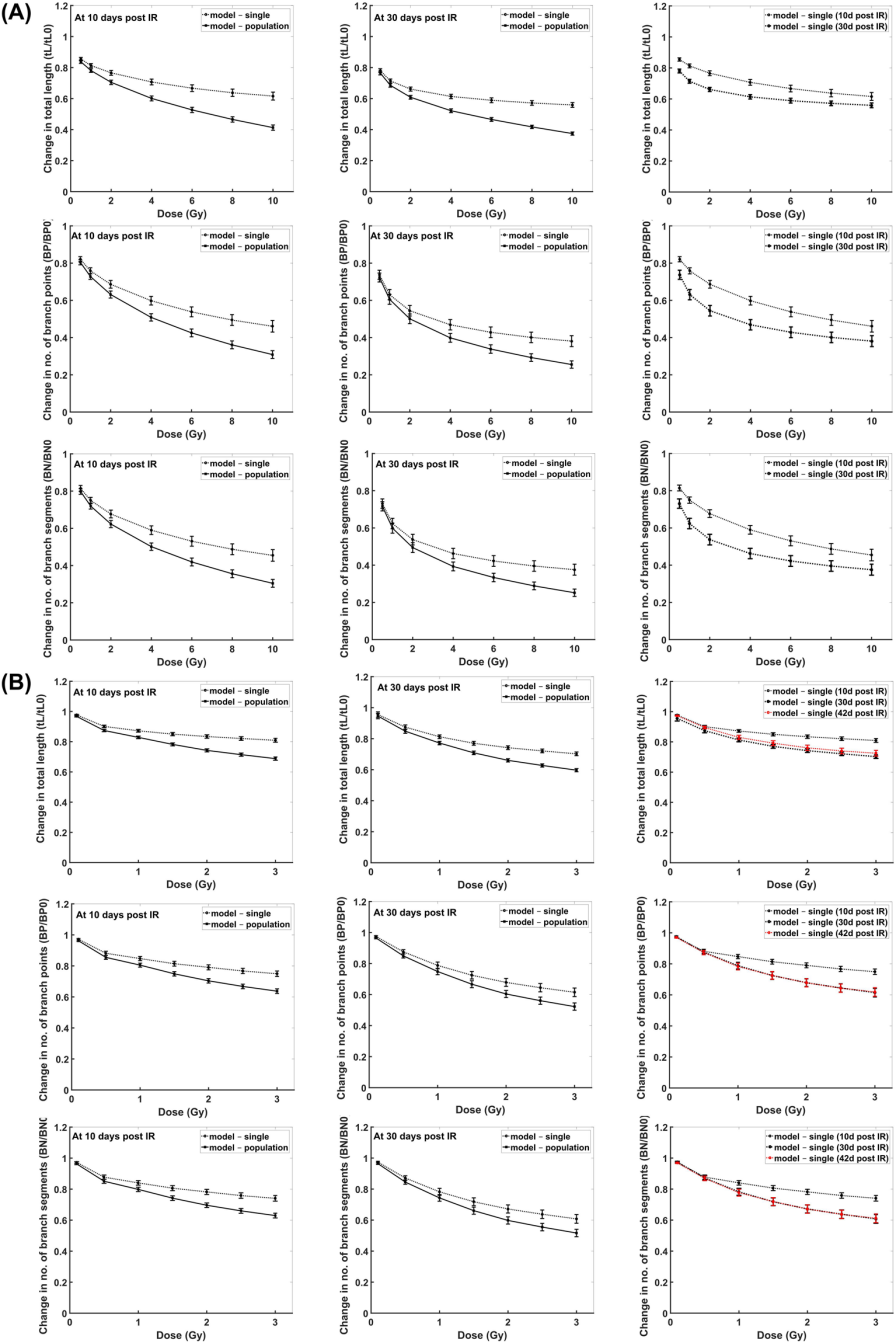
Comparison of GCL dendritic damage measurements from single neuron (dashed line) with population of neurons (solid line) induced by (**A**) gamma rays and (**B**) proton radiation at different post exposure times. (Left) single vs. population at 10 days, (Middle) single vs. population at 30 days, (Right) single at diff post exposure times.

Modeling results of dendritic damages obtained from imaging single neurons versus populations of neurons and the time-dependent dendritic damages are shown in Fig. 5. Significant dendritic damage between single neurons and populations of neurons are revealed for γ-ray doses >1 Gy and proton radiation doses >0.5 Gy at 10 days post irradiation, while γ-ray doses >2 Gy and proton radiation doses >1 Gy at 30 days post irradiation. Moreover, dendritic damage of single neuronal measurements induced by γ-rays are significantly different at 10 days and 30 days post irradiation for a dose as low as 0.5 Gy, while damage caused by proton radiation is only significantly different at 10 days and 30 days post exposure time for doses >1 Gy. Also, similar dendritic damage is manifested from 30–42 days after exposure to proton radiation.

**Figure 6:**
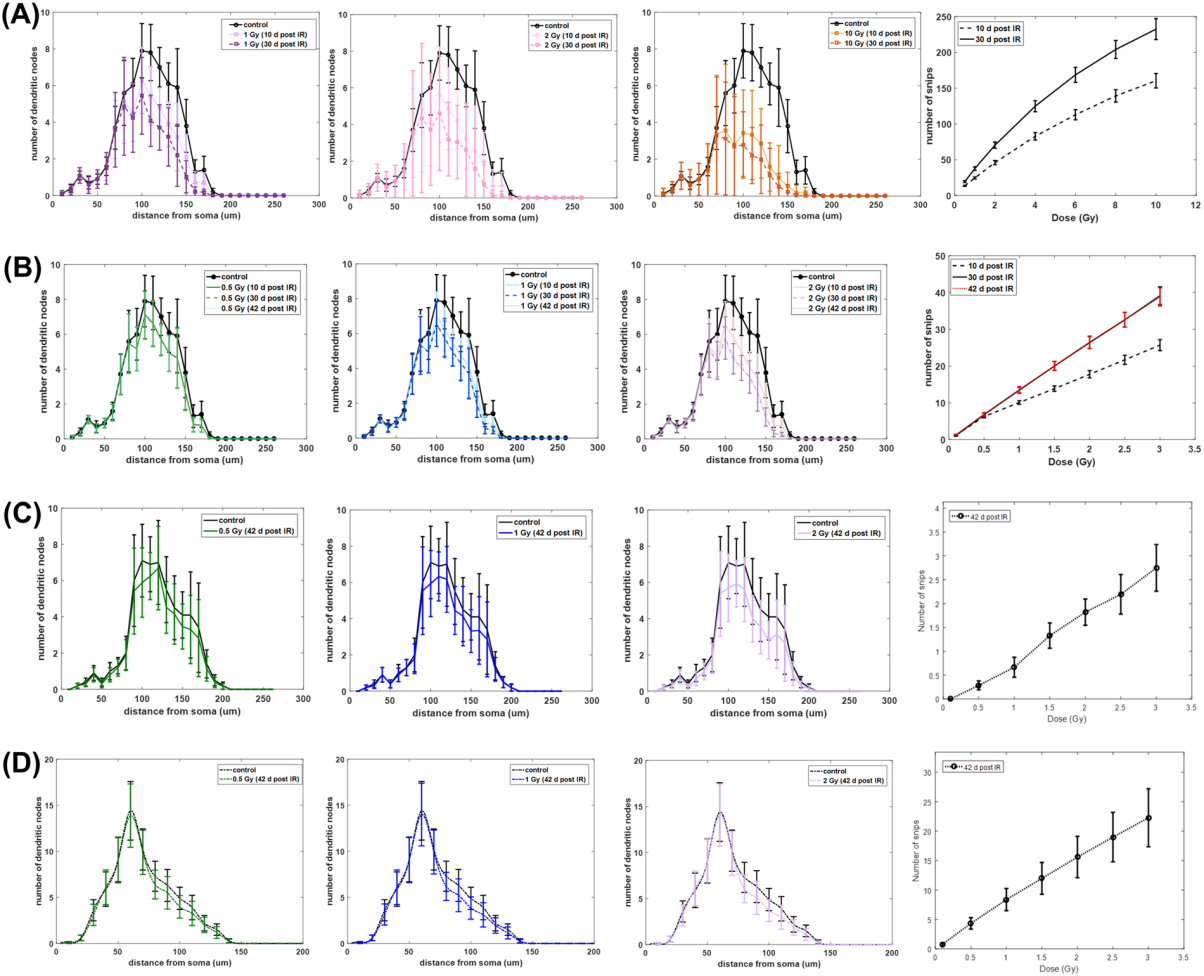
Sholl analysis and dose-dependent snip distribution of GCL neuron (**A**,**B**) and CA1 pyramidal neuron (C,D). (**A**) GCL neuron exposed to γ-rays, (**B**) GCL neuron exposed to proton IR, (**C**) Apical CA1 pyramidal neuron exposed to proton IR, (**D**) Basal CA1 pyramidal neuron exposed to proton IR. (Error bars represent standard error of the mean).

Additional modeling results are presented in Fig. 6 for both GCL and CA1 pyramidal neurons in the form of Sholl analysis and the dose-dependent number of snips. All graphs of Sholl analysis revealed no significant differences between the unirradiated and irradiated neurons, except for 10 Gy of γ-rays on GCL neurons where it shows significant reductions in dendritic arborization between 100 μm to 150 μm from the soma.

## Discussion

Understanding the structure-function relationship of neurons is important to elucidate how alterations in dendritic structure, along with spine morphology that affects synaptic inputs and integration, can influence cognition. Studies have analyzed how the morphology of hippocampal GCL and CA1 pyramidal neurons impact their functional properties^41,46^. In this paper, we develop a model that describes the time dependent alterations in neuronal dendrites of hippocampal neurons (GCL and CA1 pyramidal neurons) induced by exposure to low LET radiation such as X-rays, γ-rays and protons. Our model consists of a probabilistic component that assessed which segments would be damaged by radiation exposure, and a mathematical constituent involving ordinary differential equation to describe the kinetics of damaged segments and determine how many segments would be repaired or snipped as a function of post irradiation time. The damage probability of a given segment is dependent on radiation dose and neuronal segment volume. We associate the energy deposition of ionizing radiation with the parameter D_d_ that depends on the segment volume (V_s_). We assumed that D_d_ is defined by the Hill-type function, which provides a way to quantify the degree of dependency of D_d_ on V_s_ through the Hill coefficient (η) with a saturation dose equivalent to parameter D_m_. Moreover, we assume that each dendritic segment is discrete, thus, ordinary differential equations describing the kinetics of damaged segments are stochastically solved using the Gillespie algorithm^47^. Difference in parameter estimates of Table 1 for γ-rays and protons suggest protons are more effective which is likely due to differences in microscopic energy deposition, which includes a component from nuclear recoil nuclei and neutrons. In addition, filopodia and immature dendritic spine structures, where most excitatory synapses occur^48^, have been reported to be altered by radiation^14–17^, therefore, might affect radiosensitivity of dendrites. Our current model did not take into consideration dendritic spine structures and density in determining dendrite radiosensitivity. Future work will consider radiation effects on spine stability and the possibility that reductions in spine density influence dendritic morphology.

In the experiments considered^15,17,19^ a small number of mice per group (4 to 6) possibly leading to inter-animal variability in neuron responses. In Fig. 2, simulated neurons represent hippocampal granule cell and CA1 pyramidal neurons of young adult mice, which is the typical mouse age (1 to 4 months) used in experimental studies for radiation-induced neuron damages. Furthermore, less variability in neuron morphometric parameters were observed in young adult mice as final steps in brain development occur at 20–30 days post conception^49^. *In silico* neurons shown in Fig. 2 are generated using estimated parameters (α, β, L_i_, total dendritic length, etc.) based on neuron morphometric specifications reported in young adult mouse experiments. Our dendritic growth model can be used to simulate neurons of different age of mouse models by modifying these estimated parameters.

As presented in Figs 3 and 4, our model accurately recapitulates the dendritic morphological changes caused by exposure to low LET radiation. These modeling results have utilized experimental data derived from measurements of imaged neuronal populations^15,17^ or single neurons^19^. Due to the role of radiation-induced neuronal death^43–45,50^, our model predicts significant differences in measurements from imaging populations of neurons from brain tissue slices in contrast with single neuron imaging. These differences occurred at distinct radiation doses that depend on the type of radiation and post irradiation times. Note that in Fig. 5B, dendritic morphological changes at 30 and 42 days post irradiation are very similar. This is due to our assumption that all damaged segments would be repaired or snipped at about 30 days after radiation exposure. For future work, we can incorporate the delayed damage induced by activated microglia^10,11,51,52^ to have a more precise description of morphological change at more protracted times after irradiation.

Sholl analysis is a valuable tool to identify morphological characteristics of a neuron through dendritic arborization. Moreover, this analysis tool is also helpful in providing information useful in deciphering the mechanism/s responsible for the remodeling of neuronal structure caused by any agent^53^. For instance, pyramidal neurons have two main dendritic tree domains, apical and basal, which have different dendritic arborization patterns as delineated by Sholl analysis. Apical and basal dendrites have distinct synaptic inputs, excitability and modulation, although the degree and extent with which they function differently with one another and to other dendritic domains remains unclear^54,55^. Synaptic inputs on different dendritic domains or locations can be integrated differently to influence a particular neural activity related to certain cognitive outcomes^54^. Stress is known to cause morphological alterations in apical dendrites but not in basal dendrites of hippocampal pyramidal neurons^53,56,57^. Specifically, chronic immobilization stress reduces dendritic arborization of CA3 apical dendrites from 100 μm to 250 μm distance away from the soma^57^. In our radiation-induced dendritic damage model, arborization of CA1 apical dendrites appear to decrease from 80 μm to 140 μm from the soma (Fig. 6(C)), although not significantly, a finding that may well change following higher radiation doses. While speculative at present, this example does show the potential utility of our model in predicting different patterns of morphological alterations caused by radiation compared to other stressors or severing agents.

Another important factor that might affect radiation-induced changes in neuronal dendrites is the age of mouse models. Alterations in dendritic morphology, along with cellular connectivity, gene expression, ion potential dysregulation and other factors that may alter network connectivity and dynamics of neuron are shown to be correlated with age-related cognitive and behavioral dysfunction^58^. Furthermore, young mice have more active neurogenesis, a process that diminishes significantly with age^22,23,59–61^. Developing dendrites of adult-born neurons undergo pruning to attain homeostasis with neurons of similar dendritic structure^62^. Radiation sensitivity typically decreases with age as dividing cells and cell undergoing active metabolic processes are typically more sensitive. However, less is known about the dependence of the radiation sensitivity of dendrites with age. Along with dendritic “snipping” caused by radiation exposure, the possibility that more damage might be observed in neurons undergoing active pruning at younger ages should be considered. Nevertheless, the age-dependence of radiation-induced dendritic damage can be included in our mathematical model by modifying parameters of the characteristic dose (D_d_) in equation (4) such that apparent parameters K and D_m_ can depend on dendrite age and/or by adding a term in equations (5), (6) and (7) with parameters that represent “active pruning” at younger ages. Due to lack of experimental data showing radiation sensitivity of different neuron ages, we opt not to include “age” of neurons in our current model.

In our model, we assumed that radiation-induced changes in neuronal morphology are caused by “snipping” via dendritic fragmentation. There are two widely known cellular mechanisms of dendritic pruning: branch retraction and local degeneration or fragmentation that have been observed in drosophila^63^ with less known in rodents. The latter was observed to be the mechanism in proximal dendrites while the former occurred in distal branches and in proximal dendrites after fragmentation. Both mechanisms involved destabilization of microtubule cytoskeleton after the severing event, followed by microtubule thinning and then phagocyte-aided fragmentation and/or retraction^63^. The mechanism of radiation-induced damages in dendrites has not been established. We considered in our model that “snipping” through fragmentation is the damage mechanism (time-dependent) induced by radiation since minimal model parameters is required for this mechanism in contrast to retraction mechanism, which would require retraction rate related parameters. Future considerations in modeling damage mechanism by retraction can be made once experimental data is available.

In conclusion, we have developed an *in silico* model that describes changes in dendritic morphometric parameters induced by low LET radiation and that can also predict different patterns of morphological change compared to other stressors or dendritic damaging-agents (e.g. neurodegenerative diseases, chemotherapeutic drug, radiation) through Sholl analysis. Microdosimetric models of segment energy deposition spectra developed to consider heavy ion irradiation^64–66^ will be considered for future work, and compared with the results obtained using average segment dose that are presented in this paper.

## Methods

### Dendritic growth model

Computer modeling of neuronal morphology is a useful tool to understand structure-function relationships and recognize the role of structural changes in producing functional deficits in the brain. We have developed an *in silico* three-dimensional representation of dentate granule cell neurons in the hippocampus. Neuronal dendritic trees and branching patterns are formed with the following assumptions and morphometric determinants: (1) dendritic trees are defined by number of segments, branch points and total lengths, and are constrained to fit into a specified volume, (2) elongation and branching of individual dendrites are described as stochastic processes where probability of branching is a function of the distance grown from the soma or from the previous branch point, (3) diameter of dendrites are continuously decreasing for every elongation and branching step, and (4) isoneuronal avoidance of new fragments or growing segment is considered (24).

To generate *in silico* neurons, cylindrical branches are grown stochastically from the neuron cell soma with an initial radius of 3 μm and segment step of twice the radius (cylindrical aspect ratio of 1:1). Each step can either undergo elongation or branching, and we have assumed that probability of branching (P_br_) of each dendritic branch is described by the exponential function:

1 $${P}_{br}=\alpha [1-exp(-\beta \,\ast \,{L}_{i})]$$

where L_i_ is the distance or segment length from the soma or previous branch point, α and β are parameters that characterized a specific branching probability. For our simulation, we have assumed that hippocampal neurons (granule cell and pyramidal neurons) have the same parameters as in mouse cerebral Purkinje cells^26,28^. We used parameter β equal to 0.264^28^ while parameter α varies from 0.1 to 0.3 depending on the branch order to be consistent with the reported experimental morphometric parameters in mouse hippocampal neurons. Furthermore, dendritic radius is continuously decreasing for every elongation or branching step until it reaches 0.2 μm at the dendritic tips. Decrease in dendritic radius for each elongation step is defined by a taper rate and we assumed that mouse hippocampal granule cell layer neuron has the same taper rate as in rat hippocampal pyramidal neuron^36^. On the other hand, we defined the decrease in dendrite radius for every branching using the relationship:

2 $${R}_{p}^{2}={R}_{d1}^{2}+\,{R}_{d2}^{2}$$

where R_p_ is the parent dendrite, and R_d1_, R_d2_ are the daughter dendrites^27,37^. We have assumed that diameters of daughter dendrites after branching are the same, such that R_d1_ = R_d2_ = R_d_.

One unique feature of our *in silico* neurons is that each dendritic segment and branch, and each branch point has a unique index or identification (ID) number, which enables us to monitor changes in neuronal dendritic structures that might be caused by any damages.

### Neuronal dendritic structure after exposure to radiation

Changes in neuronal dendritic structure caused by exposure to low linear energy transfer (LET) radiation, such as X-rays, γ-rays and protons, is evaluated using average segment dose. Radiation-induced dendritic damages are expressed in Sholl analysis and as fraction of irradiated over unirradiated (X/X_0_) morphometry parameters, such as total dendritic length (tL/tL_0_), number of segments (BN/BN_0_) and number of branch points (BP/BP_0_).

In our radiation-induced dendritic damage model, number of snips or snip sites on dendritic segments are stochastically determined in these steps:

1. Each dendritic segment is assessed if it is damaged after radiation exposure (IR) using a probability function that is dependent on radiation dose (D) and neuronal segment volume (V_s_).
2. Each damaged segment can either be repaired or snipped depending on the kinetics of IR-induced damaged segments. All damaged segments are arranged in increasing damage probability (P_d_).
3. The time-dependent number of snips is evaluated using the kinetics of damaged segments represented by ordinary differential equations. Damaged segments with high damage probability will have a higher priority in snipping.

The probability that a dendritic segment is damaged after exposure to low-LET radiation is described using the exponential function:

3 $${P}_{d}=1-exp(\frac{-D}{{D}_{d}})$$

where D is the average segment dose and D_d_ is the characteristic dose where 37% of the segments are undamaged. D_d_ depends on the segment volume (V_s_) and we assumed that it is defined by the Hill-type function below, with apparent parameters K and D_m_, and Hill coefficient, η:

4 $${D}_{d}={D}_{m}(\frac{{V}_{s}^{\eta }}{K+{V}_{s}^{\eta }})$$

Each damaged segment is either repaired or snipped *S*_*0*_. The number of repaired or snipped segments is characterized by the following ordinary differential equation:

5 $$\frac{d{S}_{0}}{dt}=-{\alpha }_{d}\frac{dD}{dt}+{\propto }_{R}{S}_{d}$$

6 $$\frac{d{S}_{d}}{dt}={\alpha }_{d}\frac{dD}{dt}-{\propto }_{R}{S}_{d}\,-\,{\propto }_{S}{S}_{d}$$

7 $$\frac{d{S}_{S}}{dt}={\propto }_{S}{S}_{d}$$

where S_0_, S_d_ and S_s_ represent undamaged/repaired, damaged and snipped segments, respectively, and α_d_, α_R_ and α_S_ are the damage, repair and snip reaction rate constants, respectively. For acute IR, the first term in equations (5) and (6) are not considered, with initial number of undamaged and damaged segments stochastically determined by P_d_ and initial snipped segment equal to zero. Furthermore, we assumed that each dendritic segment is discrete, therefore, the above ordinary differential equations are stochastically solved using Gillespie algorithm^47^.

Neuronal dendrite structural changes induced by radiation exposure can be experimentally monitored in several ways. Golgi staining method may be used to image individual neurons and evaluate structural changes in a single neuron^19^. A more sensitive and robust method using neurons expressing enhanced green fluorescent protein (eGFP) could monitor structural changes but experimental data are reported as population of neurons^15,17^. To convert our modeling results of structural changes from a single neuron to population of neurons, we used a factor derived from the survival of neurons represented by the exponential function:

8 $${F}_{N}=exp(\frac{-D}{{D}_{0}})$$

where *F*_*N*_ is the fraction of surviving neurons after irradiation, D is the radiation dose and D_0_ is a characteristic dose where 37% of neurons survived. Translating dendritic structural changes from a single neuron to population of neurons can be determined using:

9 $${(\frac{X}{{X}_{0}})}_{population}={F}_{N}\,\ast \,{(\frac{X}{{X}_{0}})}_{single}$$

where X and X_0_ refers to irradiated and unirradiated morphometry parameters, respectively.

### Data analysis and mathematical modeling

All figures and plots, data fitting and analysis, modeling and computer simulation of neurons are accomplished using Matlab 2016a (Mathworks, Inc.). Differential equations describing the kinetics of radiation-induced damaged segment is solved using Gillespie algorithm written in Matlab.

## Electronic supplementary material

Supplementary Information
