# Supplementary material for: Stochastic Modeling of Radiation-induced Dendritic Damage on in silico Mouse Hippocampal Neurons
Source: Sci Rep. 2018 Apr 3;8:5494. doi: 10.1038/s41598-018-23855-9 (PMC5882641; doi:10.1038/s41598-018-23855-9)
Supplement: Supplementary file 1 — Supplementary Information [file 41598_2018_23855_MOESM1_ESM.docx]

**Supplementary File for:**

**Stochastic Modeling of Radiation-induced**

**Dendritic Damage on *in silico* Mouse Hippocampal Neurons**

Eliedonna Cacao^1^, Vipan K. Parihar^2^, Charles L. Limoli^2^ and Francis A. Cucinotta^1,*^

^1^Department of Health Physics and Diagnostic Sciences,

University of Nevada, Las Vegas, NV, United States of America

^2^Department of Radiation Oncology,

University of California, Irvine, CA, United States of America

| **(A)**   | **(C)**  **** |
| --- | --- |
| **(B)**   | **(D)**  **** |

**Supplementary Figure S1.** Effects of varying different parameters on dendrite damage probability. (A – C) Variation of Hill function apparent constants K and D_m_, and Hill coefficient, η, as a function of dendrite segment radius and its effect on D_d_. (D) Dendrite damage probability as a function of segment radius at different Hill coefficient for 1 or 10 Gy radiation dose.

| **(A)**  **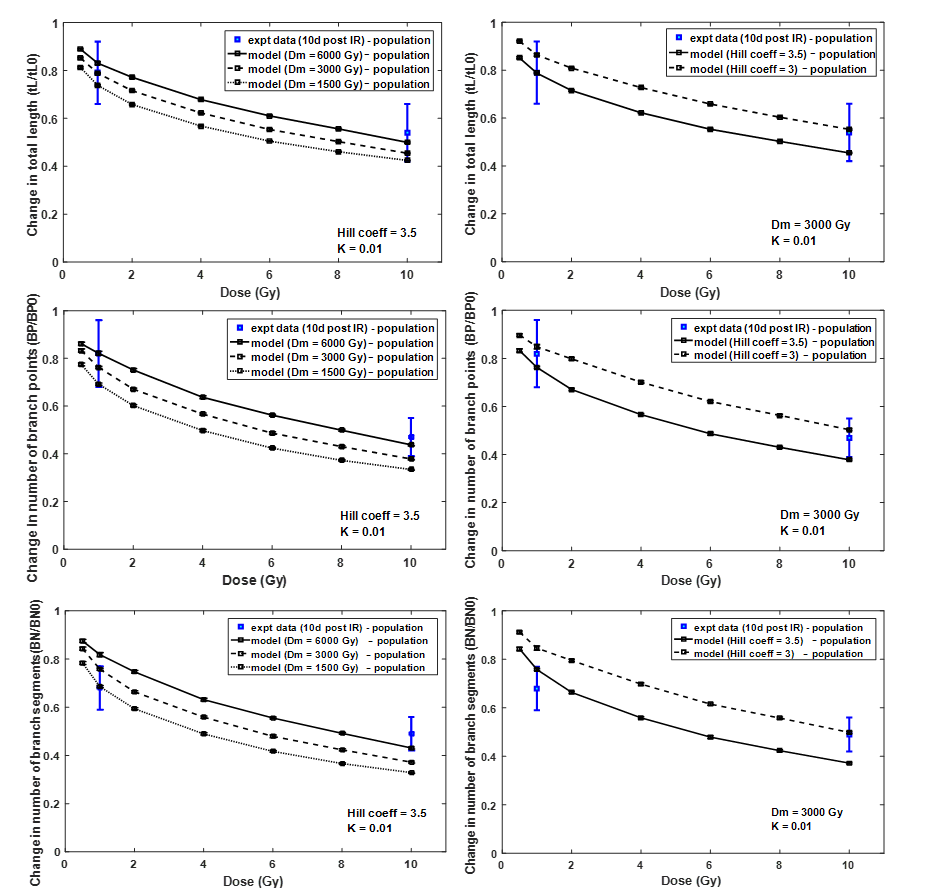** | **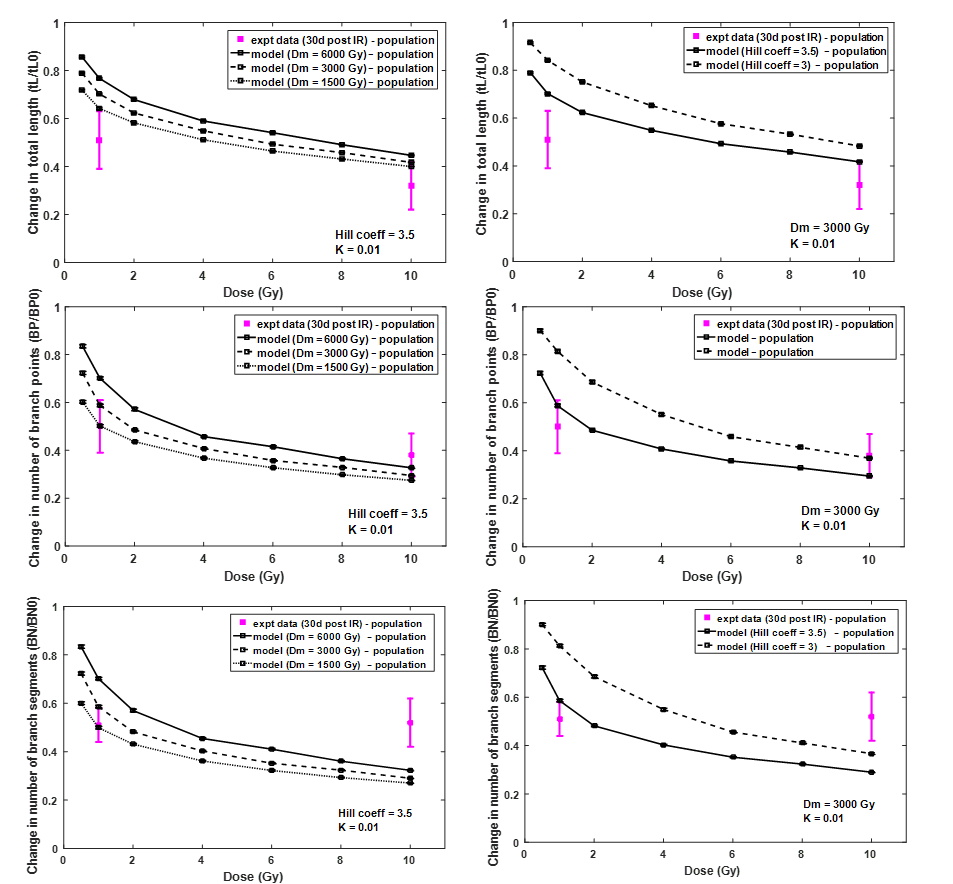** |
| --- | --- |
| **(B)**  **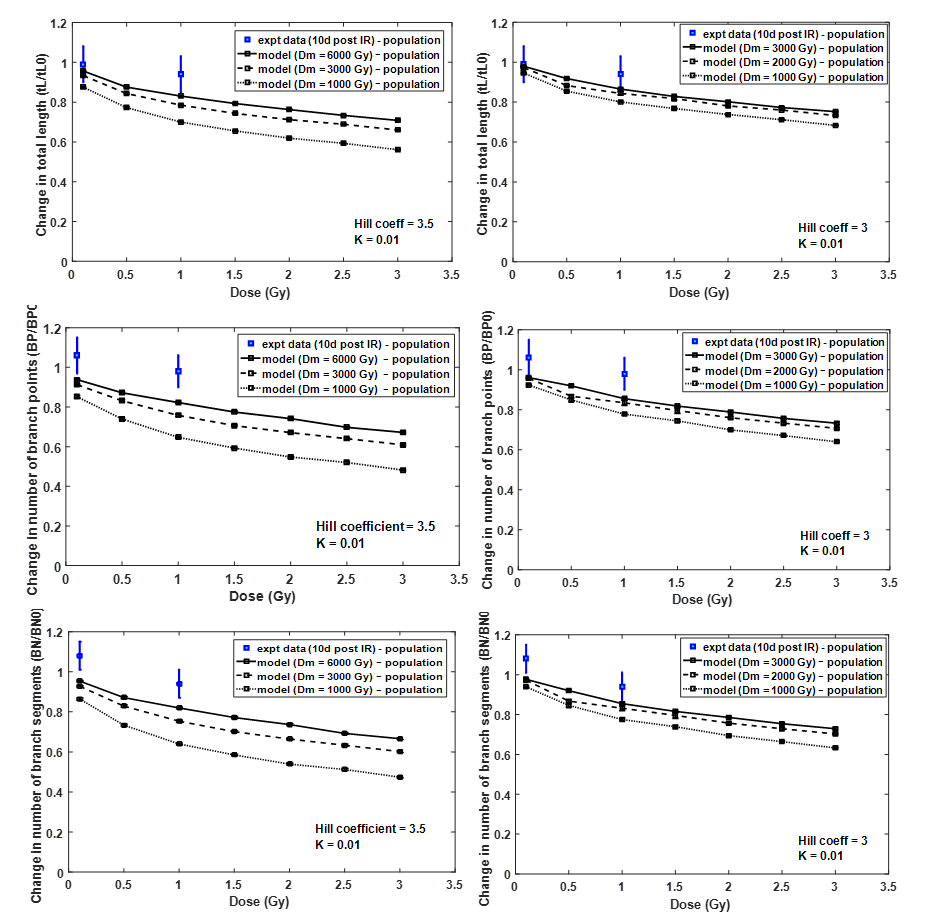** | **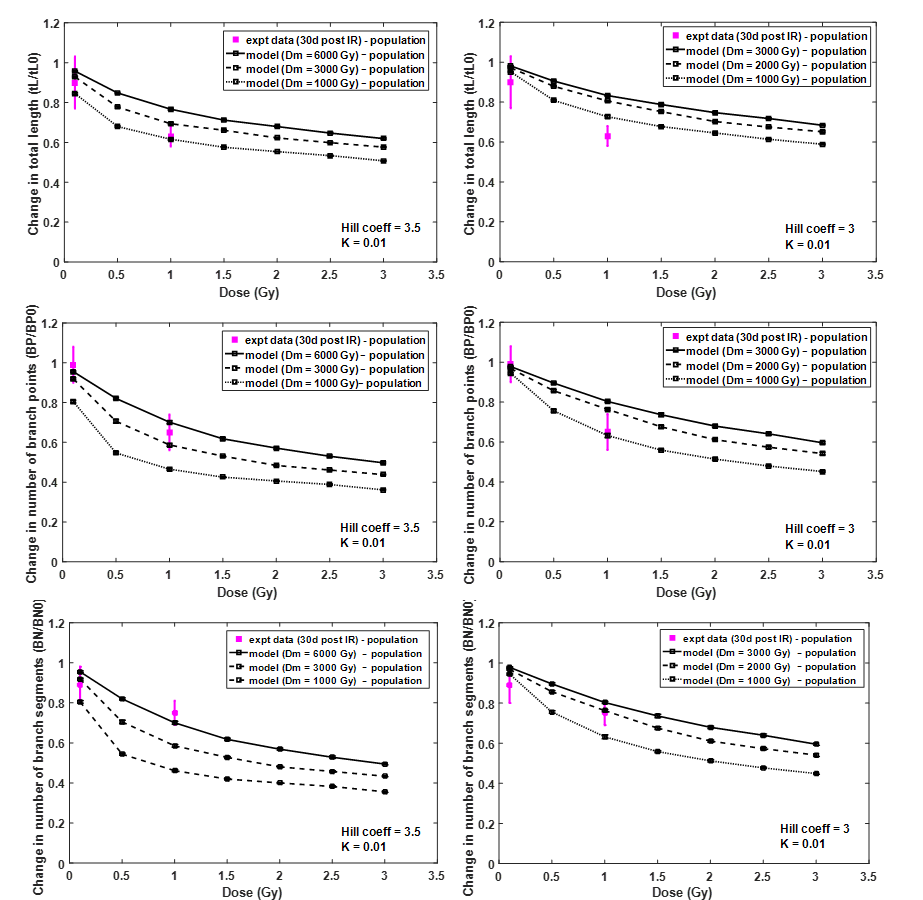** |

**Supplementary Figure S2.** Estimation of dendritic damage parameters induced by (A) gamma rays and (B) proton radiation at 10 days (blue) and 30 days (magenta) after exposure.

| **(A)**  **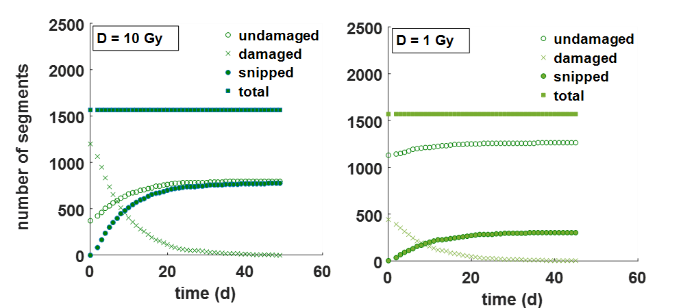** | **(B)**  **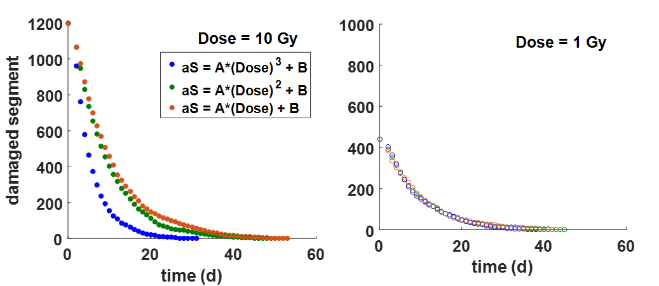** |
| --- | --- |
| **(C)**  **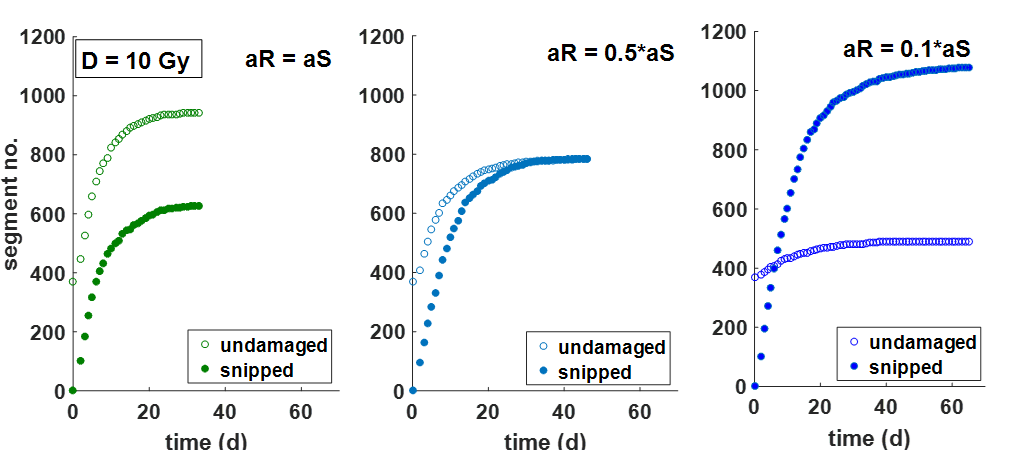**  **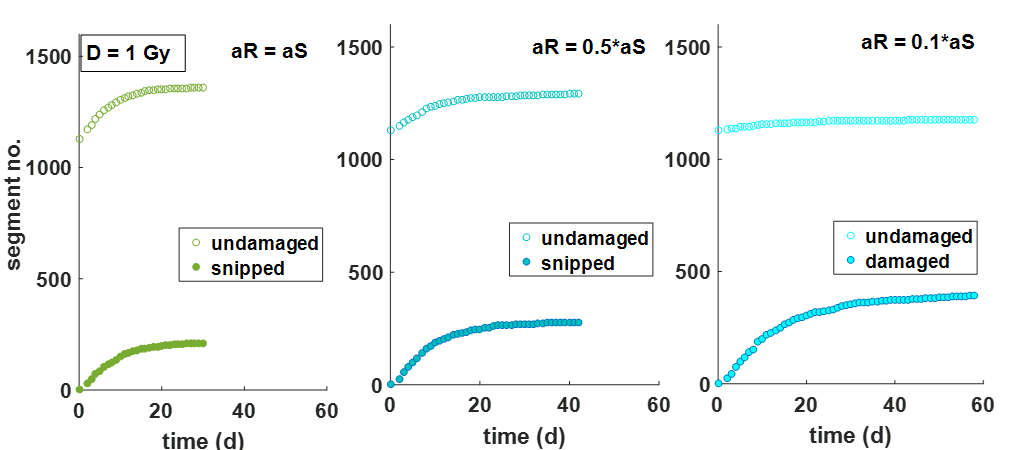** | |
| **Supplementary Figure S3.** Evaluation of parameters for kinetics of radiation-induced dendritic damage: (A) Graph showing number of undamaged, damaged, snipped and total number of segments, (B) Snip reaction rate constant as a function of dose, (C) Repair reaction rate constant as a fraction of snip reaction rate constant. | |
